# Treated Autoimmune Thyroid Disease Is Associated with a Decreased Quality of Life among Young Persons with Type 1 Diabetes

**DOI:** 10.1155/2015/185859

**Published:** 2015-05-18

**Authors:** Alena Spirkova, Petra Dusatkova, Monika Peckova, Stanislava Kolouskova, Marta Snajderova, Barbora Obermannova, Katerina Stechova, Tamara Hrachovinova, Jiri Mares, Ondrej Cinek, Jan Lebl, Zdenek Sumnik, Stepanka Pruhova

**Affiliations:** ^1^Department of Pediatrics, 2nd Faculty of Medicine, Charles University in Prague and University Hospital in Motol, V Uvalu 84, 15006 Prague, Czech Republic; ^2^Department of Psychology, Faculty of Arts, Charles University in Prague, 11000 Prague, Czech Republic; ^3^Department of Probability and Mathematical Statistics, Faculty of Mathematics and Physics, Charles University in Prague, 11800 Prague, Czech Republic; ^4^Department of Social Medicine, Faculty of Medicine in Hradec Kralove, Charles University, 50038 Hradec Kralove, Czech Republic

## Abstract

Type 1 diabetes (T1D) in children and adolescents is relatively often accompanied by other immunopathological diseases, autoimmune thyroid disease (AITD) or celiac disease (CD). Our aim was to assess whether these conditions are associated with changes in the health-related quality of life (HRQOL) in pediatric patients with T1D. In a cross-sectional study we identified eligible 332 patients with T1D aged 8–18 years, of whom 248 (75%) together with their parents responded to the PedsQL Generic and Diabetes Modules. Compared to 143 patients without thyroid autoantibodies, 40 patients with a thyroxine-treated AITD scored lower in the overall generic HRQOL (*P* = 0.014), as well as in the overall diabetes-specific HRQOL (*P* = 0.013). After adjustment for age, gender, duration of diabetes, type of diabetes treatment, and diabetes control, this association remained statistically significant for the generic HRQOL (*P* = 0.023). Celiac disease was not associated with a change in the generic or diabetes-specific HRQOL (*P* = 0.07  and  *P* = 0.63, resp.). Parental scores showed no association with AITD or celiac disease, except a marginally significant decrease in the overall generic HRQOL (*P* = 0.039) in the T1D + AITD compared to T1D group. Our study indicates that, in pediatric patients with T1D, concomitant thyroxine-treated AITD is associated with lower quality of life.

## 1. Introduction

The modern treatment of pediatric type 1 diabetes mellitus (T1D) relies not only on professional medical care but also on psychosocial support [1]. The impact of T1D on everyday life is much broader than a mere adaptation to the demanding treatment regime (balanced diet, blood glucose monitoring, insulin injections or infusion, and physical activity). Notably, the patients have been shown to experience worries of hypoglycemia or long-term complications, feelings of being different from peers, or conflicts with parents concerning limited autonomy [2–4]. The early recognition of a subgroup of children with diabetes with a decreased quality of life is of utmost importance, as it is frequently associated with an increased psychosocial distress potentially leading to a worse treatment adherence and T1D control [5, 6]. The ultimate goal of T1D control should not thus be limited to low HbA1c values and the absence of acute and late complications but must also include the subjective well-being of the children and their families.

The impact of a disease on the patients' lives is indirectly measurable by assessing their health-related quality of life (HRQOL) which is defined as “patient's subjective perception of the impact of his disease and its treatment(s) on his daily life, physical, psychological and social functioning and well-being” [7]. The generic HRQOL questionnaires focus on general aspects of quality of life and are applicable in both healthy and diseased subjects whereas the diabetes-specific HRQOL tools refer to the disease-specific impacts on daily life and well-being [8–10]. The questionnaires have also a parental version which is often administered in parallel to the patients' questionnaires.

Children with T1D often suffer from concomitant autoimmune diseases. Of these, the most common are autoimmune thyroid disease (AITD) and celiac disease (CD) with a prevalence of 15% and 4–7%, respectively [11–14]. Both diseases are readily detectable by regular screening using autoantibodies, provable by imaging techniques (AITD) or biopsy (CD), and clinical recommendations have been developed on their management in young persons with T1D [15]. While there are many studies describing the influences of treatment regime, gender, HbA1c level, and age of patients with T1D on their quality of life [16, 17], up to our knowledge, no studies have assessed the HRQOL in children with T1D and AITD and only one study has published the impact of concomitant CD in children with T1D [18].

The aim of the present work was to assess whether two most prevalent comorbid conditions (AITD and CD) were associated with changes in the quality of life in young persons with T1D, by conducting a cross-sectional study at a large tertiary centre of reference for pediatric diabetes.

## 2. Materials and Methods

### 2.1. Participants and Their Screening for AITD and CD

The setting of this study was a tertiary referral centre for pediatric diabetes at the University Hospital in Motol in Prague, Czech Republic. The centre currently provides care for 513 children and young persons with diabetes, of whom overwhelming majority have T1D. It is accredited by the ISPAD/SWEET as one of its European Centres of Reference [19].

The eligibility criteria in the present study were (i) age 8 to 18 years (age constraints imposed by the questionnaires), (ii) the diagnosis of T1D made at least one year prior to the administration of the questionnaire, (iii) normal values of TSH (even if the patient was treated for AITD): hypothyroidism and hyperthyroidism were an exclusion criterion for their known effect on the psychological status, and (iv) absence of severe chronic concomitant diseases other than AITD or CD at the time of quality of life testing. In total, participation was offered to 332 eligible patients of the centre through their parents or guardians, of whom 248 (74.6%) participated. Demographic and clinical characteristics of the participants are shown in Table 1; the study group comprised 97 children aged 8–12 years (mean 10.6 years 48.4% of girls) and 151 adolescents aged 13–18 years (mean 15.8 years 47.0% of girls). For statistical analysis, the closest level of HbA1c to the date of the questionnaires administration as well as current type of T1D treatment was recorded.

All patients are regularly screened for complications, including an annual check-up for AITD and CD. The AITD is screened using the tests for TSH levels (thyroid-stimulating hormone, normal value for both genders 0.34–5.5 mIU/L in subjects aged 1–15 years, 0.35–4.8 mIU/L in subjects aged more than 15 years), anti-thyroglobulin autoantibodies (anti-Tg, normal values 0–60.9 kU/L for both genders and all ages), and thyroid peroxidase autoantibodies (anti-TPO, normal values 0–60.9 kU/L for both genders and all ages) as described previously [13]. The patients repeatedly positive for one or both autoantibodies and with elevated TSH levels were diagnosed as having AITD which was confirmed also by typical sonographical findings in all of them. Celiac disease was screened using anti-transglutaminase IgA and/or endomysial IgA antibodies and the diagnosis was subsequently confirmed by small bowel biopsy.

The annual thyroid autoantibody screening and testing of TSH levels discriminated the children into three groups: (i) 143 children without thyroid autoantibodies (“no thyroid disease”), (ii) 65 children having positive thyroid autoantibodies (against either thyroglobulin or thyroid peroxidase, “T1D+AB” group), and (iii) 40 children who had autoimmune thyroid disease with thyroid function compensated with L-thyroxine to the normal levels of TSH (“T1D+AITD” group). It is of note that four patients did not normalize TSH levels upon treatment at the time of the study and were thus excluded from the study.

**Figure 1:**
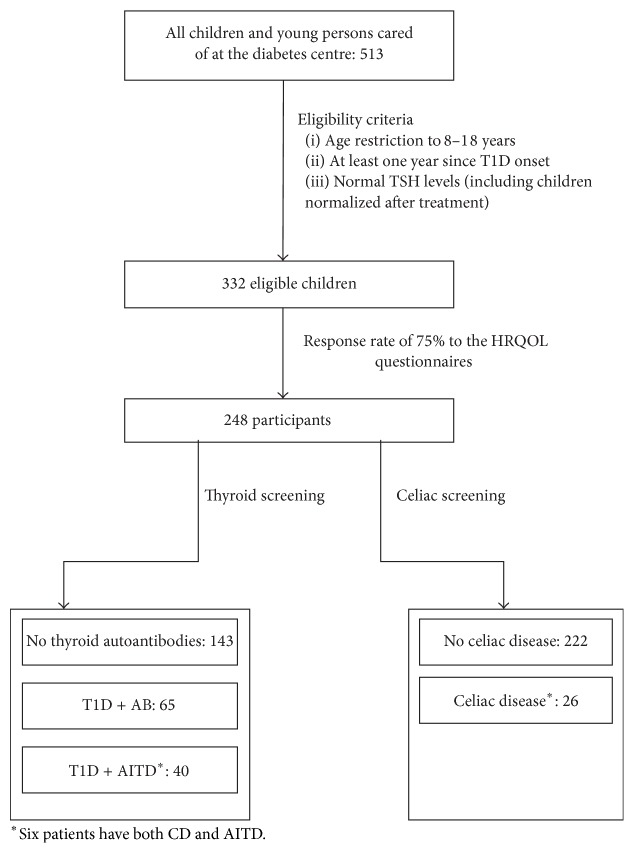
The flowchart of the study.

CD was diagnosed in 26 of 248 (10.5%) patients. All of them were treated with gluten-free diet and 16 (61.5%) were ATGA negative at the time of HRQOL testing. Six patients had both CD and AITD and were therefore included in both AITD and CD related analyses. The work flow of the study is shown in Figure 1.

### 2.2. Quality of Life Questionnaires

The study was approved by the institutional ethics committee. The participation was offered through a personal letter signed by the attending physician and encouraged during the routine outpatient visits. The two anonymous questionnaires (generic and diabetes-specific HRQOL) were filled in either at home or at the hospital before the routine visit. Questionnaires were fully completed by 225 subjects whereas in the remaining 23 patients some of the questions were left unanswered and thus excluded from the particular subanalyses. We also collected 234 complete parent proxy reports. The characteristics of children responding to the questionnaires are summarized in Table 1.

The study was carried out using the PedsQL developed by Varni et al. [20]. The generic HRQOL was assessed by the PedsQL 4.0 Generic Core Scales (acute form) instrument which contains 23 items and encompasses four dimensions: physical, emotional, social, and school functioning. Diabetes-specific HRQOL was evaluated using the PedsQL 3.2 Diabetes Module (acute form) tool consisting of 32 or 33 items (depending on the age group) divided into five dimensions: diabetes symptoms, treatment barriers, treatment adherence, worry, and communication. Patients were requested to fill in self-report and parents were asked for a parallel proxy-report form. Respondents were asked how frequently each item had been problematic for them/their children during the past week and were supposed to record their attitudes on the five-point Likert scale. Summary scores may range from 0 to 100; higher scores indicate better HRQOL. Both Czech versions of instruments have linguistic validation certificates of the MAPI Research Trust (Lyon, France) proving that the translation process was supervised and included three steps: two translations into Czech by qualified translators, following a backward translation, and eventually cognitive debriefing with three healthy children and parents (linguistic validation certificate is available upon request from the authors). The instruments have appropriate psychometric properties [21, 22] and were endorsed for this study by Alena Spirkova (personal correspondence).

### 2.3. Statistical Analysis

Differences of HRQOL between two groups (children/adolescents, boys/girls, T1D+AITD/no thyroid disease, and T1D+CD/no celiac disease) were compared by two-sample *t*-test. Welch's correction for unequal variances was performed. Differences between three groups (T1D/T1D+AB/T1D+AITD) were evaluated by *F*-test analysis of variance. Dependence of HRQOL on continuous variables (age, diabetes duration, HbA1c, and treatment) was assessed by linear model. Finally, multiple linear regression analyses were used to explore the effects of different measures to HRQOL. A *P* value less than 0.05 was considered statistically significant.

## 3. Results

### 3.1. Autoimmune Thyroid Disease

Compared to patients with T1D only, the T1D+AITD patients scored in average lower in the generic HRQOL and in the diabetes-specific HRQOL (Table 2). Significantly lower scores were observed in many dimensions, including emotional functioning and school functioning (generic HRQOL) and diabetes symptoms and treatment adherence (diabetes-specific HRQOL). Further, a borderline nominal significance was noted in physical functioning (*P* = 0.051, generic HRQOL). The T1D+AB group (patients having autoantibodies but normal thyroid function) did not differ in either of the dimensions compared to patients with T1D only. There was, however, an apparent decreasing trend in many scores in the direction from no thyroid disease through T1D+AB to T1D+AITD group (Table 2).

The analysis was then adjusted for potential confounders and modifiers such as the level of HbA1c, age, diabetes duration, gender, and type of treatment. The adjusted model, specified in Table 3, included these five clinically most relevant variables. The association of AITD with a decrease in the generic HRQOL score persisted after this adjustment (*P* = 0.023) whereas no difference was appreciable for diabetes-specific HRQOL (*P* = 0.11).

The parental questionnaires did not show alterations of scores with the exception of a decrease in the overall generic score in the T1D+AITD group (*P* = 0.039) where also borderline-significant decrease in the physical functioning was noted (*P* = 0.043).

### 3.2. Celiac Disease

Neither overall generic nor overall diabetes-specific HRQOL of patients with T1D+CD differed from T1D patients without CD; this applied to both children's and parental scores (Table 4). The only differences were observed in single dimensions: the physical functioning, in which patients with T1D+CD had higher children's scores (*P* = 0.025), and the diabetes symptoms, where patients with T1D+CD had higher parental scores (*P* = 0.020).

## 4. Discussion

We observed an independent association between AITD and a decrease in several measures of quality of life in children and adolescents with T1D, whereas no such clear association was observable for CD.

The known factors as female gender, worse glycemic control, higher age, higher diabetes duration, and type of T1D treatment only partly explained the lower scores of HRQOL in the T1D+AITD group, as well as the apparent decreasing trend of HRQOL from diabetes-only group through patients with thyroid autoantibodies to children and adolescents with treated AITD (adjusted model, Table 3). All the confounders linked to AITD in the present study, with the exception of T1D treatment, had been described before as factors related to worse HRQOL in patients with T1D [23–29].

Upon adjustment, a significant independent association of AITD with decreased HRQOL was found for generic HRQOL but not for diabetes-specific HRQOL. It could be explained by the fact that the diabetes-specific questionnaire assessed the difficulties caused particularly by diabetes whereas the generic HRQOL covers a much broader concept of main life domains (physical, emotional, social, and school functioning). Therefore, the component of the association of AITD with HRQOL, which is independent of T1D, is more apparent in this measure.

There may be several reasons why thyroid disease is linked to a decreased HRQOL. Indeed, it is well conceivable that the diagnosis of AITD further increases the burden on the patient, having another disease requiring daily substitution treatment, although with tablets only. Two dimensions were impaired: the emotional and school functioning. We can only speculate whether there may be even a direct causative relation with AITD; for the emotional dimension, there could be a mechanism involving a direct impact of thyroid hormones on serotonin neurotransmission and subsequently on the mood [30]. The school functioning might be affected by an association of thyroid function with cognitive disturbances [31].

### 4.1. Comparison to Previous Studies

We are not aware of a previous study on HRQOL in patients with AITD and diabetes, which is moreover pediatric. Several studies from adult patients without diabetes indicate that the burden of AITD is not as benign as it may seem to a healthcare professional knowledgeable about its relatively low overall health risks. It seems that the autoimmunity may affect the quality of life independent of thyroid function status: an impaired psychological well-being linked to altered quality of life was observed in treated patients with overt hypothyroidism with TSH in normal range [32–36] as well as in adults with untreated subclinical hypothyroidism [37]. Controversies exist regarding the prevalence of anxiety and depression in population with subclinical hypothyroidism: it was increased in some [38, 39] but not all studies [40]. Moreover, Ott et al. published a study where AITD had an impact on quality of life in adult women independently of their hormonal status [41]. The overall picture indicates that the AITD* per se* is a factor that may aggravate the psychological status of the patient which is in accord with the results of our multivariable model.

Interestingly, the other studied concomitant disease, CD, was not associated with worsening of HRQOL when compared to children and adolescents with T1D only. Apart from the considerably lower power to disclose such an association (only 10.5% of patients with T1D had CD), several other explanations for nonsignificant findings can be offered. First, gluten-free diet in patients with T1D+CD may have integrated into their diabetes treatment regime and therefore the awareness of dual diagnosis did not impair their HRQOL. Similar outcome was recently observed by Sud et al. [18], with a little difference, namely, that parents of children with dual diagnosis reported lower social dimension of generic HRQOL than parents of children with T1D.

Second, in children without diabetes, treated CD does not seem to decrease the quality of life total scores [42–45] although these results are not universal [46, 47]. The situation may be different in adults where a work on the dual diagnosis of T1D and CD showed a considerable negative impact on the diabetes-specific quality of life domains (diabetes related worry and social/vocational worry) as compared to adult patients with only T1D [48]. Conversely, we observed a mild, although not statistically significant, trend toward better HRQOL in T1D+CD group.

Finally, as 46% of patients with T1D+CD were using insulin pump compared to 36% of subjects with T1D only (Table 1), we adjusted the analyses for this factor: the type of treatment had been shown to affect the HRQOL in patients with T1D [49–51]. No net effect of the type of treatment was observed in our dataset, and the type of treatment did not modify the effects of the concomitant disorder on the HRQOL. Thus, further research of adequately sized longitudinal cohorts of pediatric patients with T1D+CD has to be conducted in order to clarify the herein observed subtle changes in HRQOL.

### 4.2. Parental Scores

Parental point of view did not show significant differences among studied groups with only few exceptions. The most apparent was the difference in generic HRQOL between T1D and T1D+AITD group, which well paralleled the children's questionnaires. Generally, parents assessed their child's HRQOL worse compared to their children themselves, which is in line with data from elsewhere [26].

### 4.3. The Strengths and Limitations of the Present Work

Our study, conducted at a single large pediatric centre, has the advantage of the homogeneity in language, in diabetes education, and in treatment targets and procedures. This may have rectified some of the difficulties which would inevitably arise with a bigger, yet more heterogeneous, population. Among the limitations, the most important one is the cross-sectional design which does not allow causal inference. Our findings warrant a follow-up in a longitudinal cohort to clarify the interesting contrast between the factual low disease severity of AITD and its problematic perception by the patients with T1D and parents. Secondly, the relatively low count of the individuals with a concomitant immunopathological disease limits the power to detect more subtle changes in the quality of life and does not allow meaningful investigation of the individual subdimensions of the scores.

## 5. Conclusions

Our study shows a decrease in quality of life measures in children and young persons with T1D, associated with the concomitant diagnosis of AITD. This association is partly independent of the known confounders as poor diabetes control, higher age, longer diabetes duration, and female sex and might draw attention to a specific group of patients with bigger need of (not only psychological) medical management by healthcare professionals caring for children and their families.

## Figures and Tables

**Table 1:** Characteristics of respondents.

	All patients	Thyroid disease status			Celiac disease status	
		No thyroid disease	Autoantibodies + normal TSH (T1D + AB, untreated)	Autoantibodies + high TSH upon diagnosis (T1D + AITD, treated)	No celiac disease	Celiac disease (T1D + CD)
*n*	248	143 (57.6%)	65 (26.2%)	40 (16.1%)	222 (89.5%)	26 (10.5%)

Females (*n*, %)	118 (47.6%)	63 (44.0%)	27 (41.5%)	28 (70.0%)^a^	104 (46.8%)	14 (53.8%)
Age (years)	13.7 (11.6–16.6)	13.3 (11.3–16.0)	14.0 (10.9–16.7)	15.2 (13.2–17.2)^b^	13.4 (11.7–13.9)	13.4 (11.7–13.9)
T1D treatment						
Insulin pen	156	90	39	27	142	14
Insulin pump	96 (38.7%)	53 (37.0%)	26 (40.0%)	13 (32.5%)	80 (36.0%)	12 (46.1%)
HbA1c (mmol/mol IFCC)	68 (60–77)	67 (60–75)	66 (60–76)	75 (63–82)^c^	68 (60–77)	72 (60–83)
Diabetes duration (years)	6.3 (3.0–9.3)	5.8 (2.8–9.3)	5.7 (2.8–8.8)	7.8 (5.2–10.6)	5.9 (2.8–9.3)	7.6 (4.9–9.1)

Data are described as median (interquartile range) or *n* (%).

^a^ *P* = 0.0065 as compared to subjects with no thyroid disease; ^b^ *P* = 0.0017 as compared to subjects with no thyroid disease; ^c^ *P* = 0.044 as compared to subjects with no thyroid disease.

**Table 2:** Quality of life assessed using the HRQOL questionnaires, by thyroid disease.

	All patients	Thyroid disease status			*t*-test *P* value	
		No thyroid disease	T1D + AB	T1D + AITD	T1D + AB versus no thyroid disease	T1D + AITD versus no thyroid disease
Children's scores						
Generic HRQOL questionnaire						
Overall	82.51 (10.81)	83.57 (10.47)	82.64 (10.93)	78.55 (11.17)	0.567	0.014^**∗**^
Physical functioning	87.93 (10.58)	88.47 (10.35)	88.91 (10.21)	84.45 (11.48)	0.777	0.051
Emotional functioning	74.43 (18.57)	76.67 (18.21)	72.75 (18.59)	69.20 (18.95)	0.161	0.030^**∗**^
Social functioning	90.92 (12.53)	91.25 (11.19)	92.03 (13.24)	88.00 (15.43)	0.682	0.220
School functioning	73.77 (16.12)	75.39 (15.64)	73.28 (16.79)	68.88 (16.03)	0.396	0.026^**∗**^
Diabetes-specific HRQOL questionnaire						
Overall	76.90 (12.24)	78.46 (12.25)	75.86 (12.15)	73.05 (11.56)	0.158	0.013^**∗**^
Diabetes symptoms	73.29 (13.71)	74.76 (14.00)	72.77 (12.83)	68.87 (13.41)	0.318	0.019^**∗**^
Treatment barriers	77.60 (17.07)	78.71 (17.64)	75.85 (16.57)	76.49 (15.86)	0.261	0.452
Treatment adherence	84.82 (13.45)	86.96 (12.10)	83.15 (15.38)	79.92 (13.34)	0.082	0.004^**∗∗**^
Worry	73.77 (21.92)	75.03 (22.14)	73.65 (21.81)	69.35 (21.28)	0.681	0.159
Communication	81.07 (19.79)	82.66 (18.70)	79.83 (20.42)	77.39 (22.30)	0.352	0.189

Parental scores						
Generic HRQOL questionnaire						
Overall	78.47 (11.20)	79.57 (11.19)	77.97 (11.22)	75.30 (10.85)	0.350	**0.039**
Physical functioning	83.55 (11.79)	84.53 (11.37)	83.40 (12.84)	80.24 (11.11)	0.551	**0.043**
Emotional functioning	71.38 (17.07)	72.94 (17.60)	69.37 (16.18)	69.19 (16.39)	0.162	0.230
Social functioning	86.86 (14.22)	87.15 (13.63)	88.38 (13.70)	83.24 (16.76)	0.557	0.198
School functioning	69.14 (16.56)	70.74 (16.52)	67.78 (17.96)	65.68 (13.70)	0.193	0.061
Diabetes-specific HRQOL questionnaire						
Overall	74.13 (12.43)	75.09 (12.95)	72.91 (12.27)	72.70 (10.64)	0.251	0.253
Diabetes symptoms	71.70 (13.15)	73.20 (13.53)	69.94 (13.04)	69.19 (11.37)	0.105	0.072
Treatment barriers	74.48 (17.62)	75.04 (18.12)	73.69 (17.42)	73.81 (16.44)	0.615	0.695
Treatment adherence	79.62 (16.11)	81.02 (15.22)	77.31 (18.70)	78.46 (14.27)	0.167	0.343
Worry	72.12 (22.13)	72.43 (22.72)	72.36 (22.84)	70.53 (23.92)	0.985	0.630
Communication	76.25 (23.19)	75.91 (23.32)	76.16 (22.84)	77.67 (23.92)	0.944	0.695

Data are mean (SD).

Test for trend across the categories “no thyroid disease” > “T1D + AB” > “T1D + AITD” significant at ^∗^ *P* < 0.05 and ^∗∗^ *P* < 0.005.

**Table 3:** The multivariable model describing the influence of AITD and other clinically relevant modifiers on the HRQOL.

Predictor	Change in HRQOL score per unit (95% conf. interval)	*P* value
Analysis of generic HRQOL score		
Presence of AITD	**−4.60 (−8.56, −0.64)**	**0.023**
Gender (male)	1.99 (−1.26, 5.25)	0.23
Age (per 1 year)	0.12 (−0.49, 0.74)	0.69
Diabetes duration (per 1 year)	0.21 (−0.24, 0.66)	0.36
HbA1c (per 1 mmol/mol)	−0.07 (−0.17, 0.02)	0.14
Treatment (insulin pump)	−1.68 (−5.08, 1.72)	0.33

Analysis of diabetes-specific HRQOL score		
Presence of AITD	−3.61 (−8.05, 0.83)	0.11
Gender (male)	**5.84 (2.23, 9.46)**	**0.002**
Age (per 1 year)	0.23 (−0.46, 0.92)	0.51
Diabetes duration (per 1 year)	0.13 (−0.38, 0.64)	0.61
HbA1c (per 1 mmol/mol)	**−0.11 (−0.22, −0.01)**	**0.041**
Treatment (insulin pump)	−2.38 (−6.15, 1.38)	0.21

The dependent variable was the total score in the respective questionnaire. The predictors were presence of autoimmune thyroiditis (1 = AITD; 0 = no thyroid disease), along with five major diabetes-related confounders identified from the literature as well as from our univariate analysis. The coefficients represent the change in the quality of life score per one unit of predictor (i.e., presence of AITD, sex, one year of age, one mmol/mol of HbA1c, or treatment by insulin pump). This model was selected for its biological plausibility; that is, no stepwise building was employed.

**Table 4:** Quality of life assessed using the HRQOL questionnaires, by presence of celiac disease.

	All patients	Celiac disease		*t*-test *P* value
		No	Yes	
Children's scores				
Generic HRQOL questionnaire				
Overall	82.51 (10.81)	82.19 (10.97)	85.19 (9.05)	0.128
Physical functioning	87.93 (10.58)	87.46 (10.68)	91.88 (8.91)	**0.025**
Emotional functioning	74.43 (18.57)	73.93 (18.71)	78.65 (17.18)	0.198
Social functioning	90.92 (12.53)	90.85 (12.47)	91.54 (13.25)	0.803
School functioning	73.77 (16.12)	73.67 (16.20)	74.62 (15.68)	0.774
Diabetes-specific HRQOL questionnaire				
Overall	76.90 (12.24)	76.74 (12.21)	78.30 (12.64)	0.562
Diabetes symptoms	73.29 (13.71)	72.84 (13.61)	77.00 (12.30)	0.169
Treatment barriers	77.60 (17.07)	77.83 (17.16)	75.58 (16.51)	0.516
Treatment adherence	84.82 (13.45)	84.73 (13.53)	85.54 (13.01)	0.767
Worry	73.77 (21.92)	73.64 (22.31)	74.84 (18.65)	0.769
Communication	81.07 (19.79)	81.58 (19.48)	76.72 (22.18)	0.303

Parental scores				
Generic HRQOL questionnaire				
Overall	78.47 (11.20)	78.16 (11.39)	80.88 (9.48)	0.187
Physical functioning	83.55 (11.79)	83.29 (11.97)	85.58 (10.30)	0.303
Emotional functioning	71.38 (17.07)	70.76 (17.23)	76.35 (15.14)	0.090
Social functioning	86.86 (14.22)	86.57 (14.32)	89.23 (13.39)	0.350
School functioning	69.14 (16.56)	69.06 (16.69)	69.81 (15.78)	0.822
Diabetes-specific HRQOL questionnaire				
Overall	74.13 (12.43)	73.70 (12.36)	77.62 (12.78)	0.149
Diabetes symptoms	71.70 (13.15)	70.98 (13.02)	77.57 (12.98)	**0.020**
Treatment barriers	74.48 (17.62)	74.47 (17.88)	74.54 (15.73)	0.985
Treatment adherence	79.62 (16.11)	79.27 (16.47)	82.46 (12.77)	0.253
Worry	72.12 (22.13)	71.72 (22.18)	75.44 (21.87)	0.428
Communication	76.25 (23.19)	76.33 (22.81)	75.56 (26.71)	0.891

Data are mean (SD).
